# Mice with Different Susceptibility to Japanese Encephalitis Virus Infection Show Selective Neutralizing Antibody Response and Myeloid Cell Infectivity

**DOI:** 10.1371/journal.pone.0024744

**Published:** 2011-09-16

**Authors:** Kai Wang, Vincent Deubel

**Affiliations:** Key Laboratory of Molecular Virology and Immunology, Institut Pasteur of Shanghai, Shanghai Institute for Biological Sciences, Shanghai, China; Blood Systems Research Institute, United States of America

## Abstract

**Background:**

Japanese encephalitis virus (JEV) is a mosquito-borne flavivirus that causes public health problems in Asian countries. Only a limited number of JEV-infected individuals show symptoms and develop severe encephalitis, indicating host-dependent susceptibilities.

**Methodology/Principal Findings:**

C3H/HeN and DBA/2 mice, which exhibit different mortalities when infected by intraperitoneal inoculation with JEV, were used as experimental models to compare viral pathogenesis and host responses. One hundred infectious virus particles killed 95% of C3H/HeN mice whereas only 40% of DBA/2 mice died. JEV RNA was detected with similar low levels in peripheral lymphoid organs and in the sera of both mouse strains. High levels of viral and cytokine RNA were observed simultaneously in the brains of C3H/HeN and DBA/2 mice starting on days 6 and 9 post-infection, respectively. The kinetics of the cytokines in sera correlated with the viral replication in the brain. Significantly earlier and higher titers of neutralizing antibodies were detected in the DBA/2 strain. Primary embryonic fibroblasts, bone marrow-derived dendritic cells and macrophages from the two mouse strains were cultured. Fibroblasts displayed similar JEV replication abilities, whereas DBA/2-derived myeloid antigen-presenting cells had lower viral infectivity and production compared to the C3H/HeN–derived cells.

**Conclusions/Significance:**

Mice with different susceptibilities to JEV neuroinvasion did not show changes in viral tropism and host innate immune responses prior to viral entry into the central nervous system. However, early and high neutralizing antibody responses may be crucial for preventing viral neuroinvasion and host fatality. In addition, low permissiveness of myeloid dendritic cells and macrophages to JEV infection *in vitro* may be elements associated with late and decreased mouse neuroinvasion.

## Introduction

Japanese encephalitis virus (JEV) is an enveloped virus with a genome of single-stranded positive RNA approximately 11kb in size and belongs to the genus *Flavivirus* in the family *Flaviviridae* [1]. JEV is transmitted by mosquitoes of the *Culex* species and has an enzootic life cycle involving swine and birds, and could infect a large variety of wild animals. Humans and horses are incidental and dead end hosts of the viral cycle [2], [3].

The majority of human JEV infections are asymptomatic or mildly symptomatic, where only about 1 in 250 infected persons develop a clinical disease. However, Japanese encephalitis is still the leading cause of viral encephalitis in Asia with 30,000 to 50,000 cases reported yearly to the World Health Organization and resulting in an estimated 10,000 to 15,000 deaths annually [1], [4], [5]. For patients developing a more severe form of the disease, the illness can progress to serious infection of the central nervous system (CNS) with fatal outcomes in 30% of cases [2], [4], which indicates a distinct susceptibility to JEV infection in different people. JEV is closely related to the West Nile virus (WNV) and St. Louis encephalitis virus. After infection, these viruses may invade the CNS, including the brain and spinal cord; however, the routes employed for crossing the blood brain barrier (BBB) remain unclear. Some reports have proposed possible mechanisms for flaviviruses to infect peripheral cells and organs and enter into the CNS [6], [7], [8], [9]. Previous studies have shown that JEV can replicate in leukocytes of humans and mice [10], [11], which implies that the virus may infect the CNS through peripheral inflammatory cells.

Initial signs of JEV infection are nonspecific and viremia is rarely detected, rendering studies of viral and biological markers and any correlation with disease outcomes difficult to access in humans. Early signs of innate and cellular immune irregularities toward JEV infection may be associated with the severity of the disease [12], [13], [14]. In JEV-infected patients, an increase of interferon (IFN)-α, interleukin (IL)-6, IL-8 in cerebrospinal fluid (CSF) and “regulated upon activation normal T-cell expressed and secreted” (RANTES) have also been reported [14]. Low levels of IgM and IgG were detected in both serum and CSF in patients with fatal outcomes [15].

Mice are suitable animal models of infection with JEV, which can reproduce symptoms and physiopathological markers observed in humans [16], [17], [18]. A notable increase of proinflammatory molecules including IFNs, macrophage migration inhibitory factor (MIF), IL-6, RANTES and monocyte chemotactic protein-1 (MCP-1) have been reported in mice infected with JEV; however, the strains and ages of the mice as well as the routes of viral inoculation were diverse [18], [19], [20], [21], [22]. Laboratory mice are generally susceptible to flaviviruses, due to the natural lack of the flavivirus resistance gene [23], [24], but different strains display various severities of the disease. Differences in mouse susceptibilities to JEV were observed after infection by peripheral routes [25], [26], [27], but specific markers associated with different disease outcomes have not yet been identified. In WNV infections, survival rates of different mouse strains were not related to tissue tropism and viral replication [28].

In order to identify biological and virological markers associated with susceptibilities to JEV infection, we compared two mouse strains exhibiting different mortality rates after JEV peripheral infection. Data suggest that differences in viral infection of myeloid cells leading to dissimilar ratios of JEV production and subsequent local pro-inflammatory cytokine release with dissimilarities in level of neutralizing antibody responses may account for variations observed in the timing before CNS infection and mortality.

## Materials and Methods

### Virus

The SA14 strain of JEV (a generous gift from Pr. Yongxin Yu) was propagated in BHK-21 cell line (purchased from Shanghai Institute of Biological Sciences, Chinese Academy of Sciences, Shanghai, China). Cells were infected at a multiplicity of infection (moi) of 0.1 plaque forming unit (pfu)/cell and the virus was harvested from the cell supernatant two days post infection (p.i.), but before cellular cytopathic effects. Viral titers in pfu were obtained by plaque assay using BHK-21 cell line. The virus was aliquoted and stored at −80°C. Viral inactivation was carried out by exposure to UV radiation (wavelength of 254 nm) at a distance of 3 cm for 30 min on ice. Virus inactivation was tested by the absence of immunofluorescence in BHK cells incubated for seven days with inactivated JEV.

### Mouse strains

Six-week-old female C3H/HeN and DBA/2 mice were purchased from Vital River Lab Animal Technology Co., Ltd (Beijing, China) and Shanghai Laboratory Animal Co., Ltd (SLAC, Shanghai, China), respectively. The mouse experiment protocol was approved by the Institut Pasteur of Shanghai Ethics Committee for Animal Care (ID# 01-2006) and was carried out in the Institut Pasteur animal facility, following the European directive 86/609/EEC on the protection for animal used for experimental and other scientific purposes.

### Mouse infection

Mice were kept in isolators in a biosafety-level-2 animal facility and infected with SA14. A series of SA14 virus dilutions in PBS from 0.1 pfu to 10,000 pfu in 0.2 ml was injected by intraperitoneal (i.p.) inoculation in C3H/HeN and DBA/2 mice. The morbidity and mortality were observed daily for 1 month. For pathophysiological analysis of virus infection, the mice were injected i.p. with 100 pfu. Blood and tissue samples were harvested from three anesthetized and sacrificed mice at days 1, 3, 5, 6, 7, 8, 9, 11, 13, 15, 17, 19, 21 p.i.. Mice showing symptoms were preferentially chosen.

### Primary cell isolation and viral infection

Cells were incubated with desired viral moi for 1 hr. The virus was subsequently removed and cells were washed three times with PBS and incubated in culture medium. At 6, 12, 24, 48, 72, 96 hr p.i., the supernatants and cells were collected for virus titration and RNA extraction, respectively.

Mouse embryo fibroblasts (MEF) were prepared from 14-day-old embryos and cultured in Dulbecco's Modified Eagle's Medium (DMEM) (Invitrogen, Carlsbad, CA, USA) that contained 10% fetal bovine serum (FBS) and 100 U/ml penicillin and 100 µg/ml streptomycin, at 37°C in 5% CO_2_. The cells passaged for 2 to 4 times were trypsinized, counted and then infected with the virus at moi of 0.1 pfu/cell.

Bone marrow macrophages (BMM) and bone marrow dendritic cells (BMDC) were generated as described previously [29], [30], [31]. Briefly, cells were isolated from the bone marrow of 6-week-old mice. For generating BMM, cells were cultured in DMEM for 7 days in 10% FBS and 20% L929 conditioned medium. For generating BMDC, cells were cultured in RPMI 1640 (GIBCO, Grand Island, NY) for 10 days in 10% FBS, IL-4 and granulocyte-macrophage colony-stimulating factor (GM-CSF) (PeproTech, Inc. Rocky Hill, NJ, USA). CD11c+ BMDC were further purified by MACS beads separation (Miltenyi Biotec, Auburn CA, USA). Both BMM and BMDC were infected at a moi of 1 pfu/cell.

### Quantitative RT-PCR

RNA was extracted using the RNeasy mini kit (QIAGEN, Hilden, Germany) from tissue and cell samples and by the QIAamp Viral RNA Mini Kit (QIAGEN) from sera and cell culture supernatants according to the manufacturer's instruction. The primers used to detect JEV and glyceraldehyde 3-phosphate dehydrogenase (GAPDH) RNAs and other RNA gene factors are listed in Table 1. Isolated RNA was quantified by QuantiTect SYBR Green RT-PCR kit (QIAGEN). Briefly, the reaction condition was 50°C for 30 min, 95°C for 15 min, 40 cycles of 94°C for 15 sec, 55°C for 30 sec, and 72°C for 30 sec followed by a melting curve analysis step using the ABI PRISM 7900HT Real Time PCR System (Applied Biosystems, Foster City, CA, USA).

**Table 1:** Primers of mouse genes used in the experiment for SYBR green quantitative PCR.

Gene	Forward primer	Reverse primer	Reference
JEV	5′-AGAACGGAAGAYAACCATGACTAAA-3′	5′-CCGCGTTTCAGCATATTGAT-3′	[61]
GAPDH	5′-TGACGTGCCGCCTGGAGAAA-3′	5′-AGTGTAGCCCAAGATGCCCTTCAG-3′	[62]
CD4	5′-CCCAGGTCTCGCTTCAGTTTG-3’	5′-AGGTAGGTCCCATCACCTCACAG-3′	[63]
CD8b	5′-GCTGGTAGTCTGCATCCTGCTTC-3′	5′-TTGCTAGCAGGCTATCAGTGTTGTG-3′	[63]
CD11b	5′-GACCGTCTGCGCGAAGGAGATA-3′	5′-CGCCTGCGTGTGTTGTTCTTTG-3′	[64]
IFN-γ	5′-CGGCACAGTCATTGAAAGCCTA-3′	5′-GTTGCTGATGGCCTGATTGTC-3′	[63]
IL-6	5′-CAACGATGATGCACTTGCAGA-3′	5′-TGGTACTCCAGAAGACCAGAGGAA-3′	[65]
IL-12p40	5′-GGAAGCACGGCAGCAGAATA-3′	5′-AACTTGAGGGAGAAGTAGGAATGG-3′	[66]
MCP-1	5′-TGCAGGTCCCTGTCATGCTT-3′	5′-AGCAGGTGAGTGGGGCGTTA-3′	[65]
RANTES	5′-CTGCCGCGGGTACCATGAAG-3′	5′-TACAGGGTCAGAATCAAG-3’	[66]

### Histology and immunohistochemistry

Spleen, thymus and brain tissues from mice were fixed in 4% paraformaldehyde (PFA) overnight and sectioned in paraffin. For histological examination, the tissues were stained with haematoxylin and eosin. For immunohistochemistry, sections were deparaffinized with xylene, rehydrated through successive bathes of ethanol/water (from 100% ethanol successively to 50% ethanol till pure water) and incubated in 3% H_2_O_2_ at room temperature. The sections were then put in 10 mM sodium citrate buffer for 1 hr at 96°C for antigen retrieval and blocked with BSA at saturation for 20 min. Polyclonal rabbit anti-JEV envelope (E) antibody C0859 (produced in the laboratory against highly purified recombinant E protein) was applied to the section overnight at 4°C. Horse radish peroxidase-conjugated anti-rabbit polyclonal antibody was used as a secondary antibody and 3′-Diaminobenzidine as a chromogen (DAKO, Glostrup, Denmark). The section was counterstained with haematoxylin.

### Immunofluorescence

After 48 hr of infection, the cells were fixed with 4% PFA and permeabilized with permeabilization buffer (Triton X-100 0.1% in PBS). After washing, the cells were incubated with anti-JEV E polyclonal antibody for 1.5 hr followed by blocking with 5% BSA in PBS. Alexa Fluor 488 goat anti-rabbit antibody (Invitrogen, Eugene, OR, USA) and Hoechst 33258 (Sigma, St. Louis, MO, USA) were applied to stain the viral E protein and the cell nucleus, respectively. The slides were mounted and the images were visualized by fluorescence microscopy (Leica, Heidelberg, Germany).

### Flow Cytometry

The mock or infected BMM were trypsinized and washed with flow cytometry staining buffer (eBiosciences, San Diego, CA, USA). After blocking with anti-mouse CD16/32, the cells were incubated with alexa fluor 488 conjugated anti-mouse F4/80 (eBiosciences) for 45 min at 4°C in the dark. The surface stained cells were fixed by PFA, resuspended in permeabilization buffer (eBiosciences) and then incubated with C0863 anti-JEV NS1 rabbit polyclonal antibody (produced in the laboratory against highly purified recombinant NS1 protein) for 1.5 hr. After washing, the alexa fluor 633 conjugated anti-rabbit IgG secondary antibody (Invitrogen) was added. Fluorescence was measured using a LSRII flow cytometer (BD biosciences, San Jose, CA, USA).

### Cytokine array

The cytokine change in the sera of infected mice was tested using a Q-Plex™ Mouse Cytokine array (16-plex, Quansys Biosciences, Logan, UT, USA.) according to the manufacturer's instructions. Briefly, the sera and antigen standards were diluted and incubated for 1 hr at room temperature. After the substrates were added, images were acquired using a FUJI LAS400mini camera and analyzed with Quansys Q-View™ Software.

### Plaque reduction neutralization test

The titers of neutralizing antibodies against JEV in mouse sera were determined by the plaque reduction neutralization test (PRNT) as described previously [32]. Briefly, after inactivation of the virus and complementin the sera by heating at 56°C for 30 min, two-fold serial dilutions of the samples in DMEM, beginning with a dilution of 1∶20, were incubated with 100 pfu of JEV SA14 for 1 hr at 37°C. The mixture was incubated for 1 hr with sub-confluent BHK cells in 24-well culture plates and recovered with 2 ml of 1% carboxy methyl cellulose in DMEM containing 3% FBS. After 4 days of incubation, the cell supernatant was removed and cells were fixed and stained in a mixture of 0.1% amidoschwarz and 25% isopropanol, 10% acetic acid. The PRNT titers were calculated as the serum dilution that reduced by 50% the number of pfu compared to the tests carried out by using sera of mock-infected mice.

### Statistical analysis

The statistical analysis was performed using SAS6.12 software (SAS Institute Inc., Cary, NC, USA). Two-tailed t-tests were used to determine the significant differences and a p value less than 0.05 was regarded to be statistically significant. Two-way analysis of variation (ANOVA) was used to compare the two mouse strains at different time points.

## Results

### Individual mouse strains show selective susceptibilities to infection with JEV

**Figure 1:**
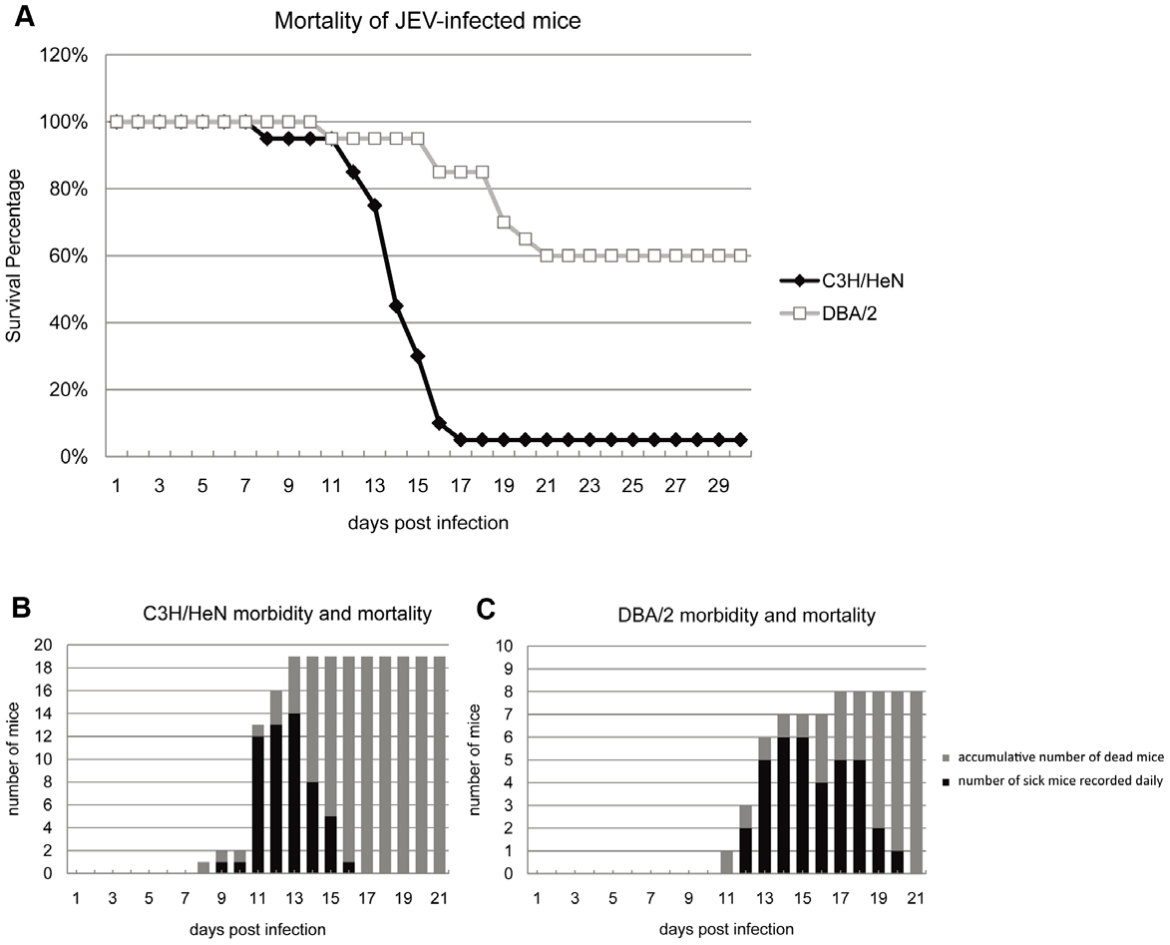
Mortality of C3H/HeN and DBA/2 mice after intraperitoneal infection with JEV. Twenty mice of each C3H/HeN and DBA/2 strain were infected intraperitoneally with (100 pfu) of JEV strain SA14 and were observed for four weeks. The percentage of surviving C3H/HeN (open square) and DBA/2 (black triangle) mice after infection was recorded (A). The number of sick mice was recorded daily (black column) and the cumulative number of death is indicated (grey column). All remaining mice with no symptoms after three weeks survived (B-C).

Mice with different haplotypes have shown dissimilar susceptibilities to JEV infection [25], [26], [27]. In the present study, 20 six-week-old mice of H2^k^ (C3H/HeN) and H2^d^ (DBA/2) haplotype strains were shown to be the most and the least susceptible to i.p. doses of JEV strain SA14 ranging from 1 to 10,000 pfu, respectively (data not shown). For the remainder of the experiments, mice of both strains were infected with 100 pfu. In total, 19 C3H/HeN mice (95%) died by day 17, where only eight DBA/2 mice (40%) died by day 21 (p = 0.0013) (Fig. 1A). Deaths of C3H/HeN mice began on day 8 and 3 days later in DBA/2 (Fig. 1). Symptoms including rough fur, hunching of the back and hind limb paralysis appeared around 2 to 3 days before total paralysis, convulsions and death in both mouse strains (Fig. 1B, C). Time laps between symptoms apparition and death was longer in DBA/2 (Fig. 1B, C). After 3 weeks, all surviving mice showed no symptoms and were sacrificed at 30 days p.i.

In contrast, intracerebral inoculation of JEV caused more rapid and earlier deaths by day 6 p.i. in both strains and at the same rate (data not shown), suggesting that the difference in susceptibilities of mice towards JEV infection was likely due to virus-host interactions before viral entry into the brain.

### Virus load in tissues of JEV infected mice

RNA was extracted from sera of three mice at different time points p.i. and the number of JEV copies was quantified by real-time RT-PCR by comparison with a standard curve drawn from titrated JEV RNA transcripts (data not shown). Sick mice were preferentially chosen for sampling at indicated days. Briefly, other than one C3H/HeN mouse picked on day 8 and another on day 9 p.i., all the mice sampled after day 11 p.i. showed symptoms of JEV infection. Only two DBA/2 mice sacrificed at day 11 p.i. and one at day 13 p.i. were sick.

**Figure 2:**
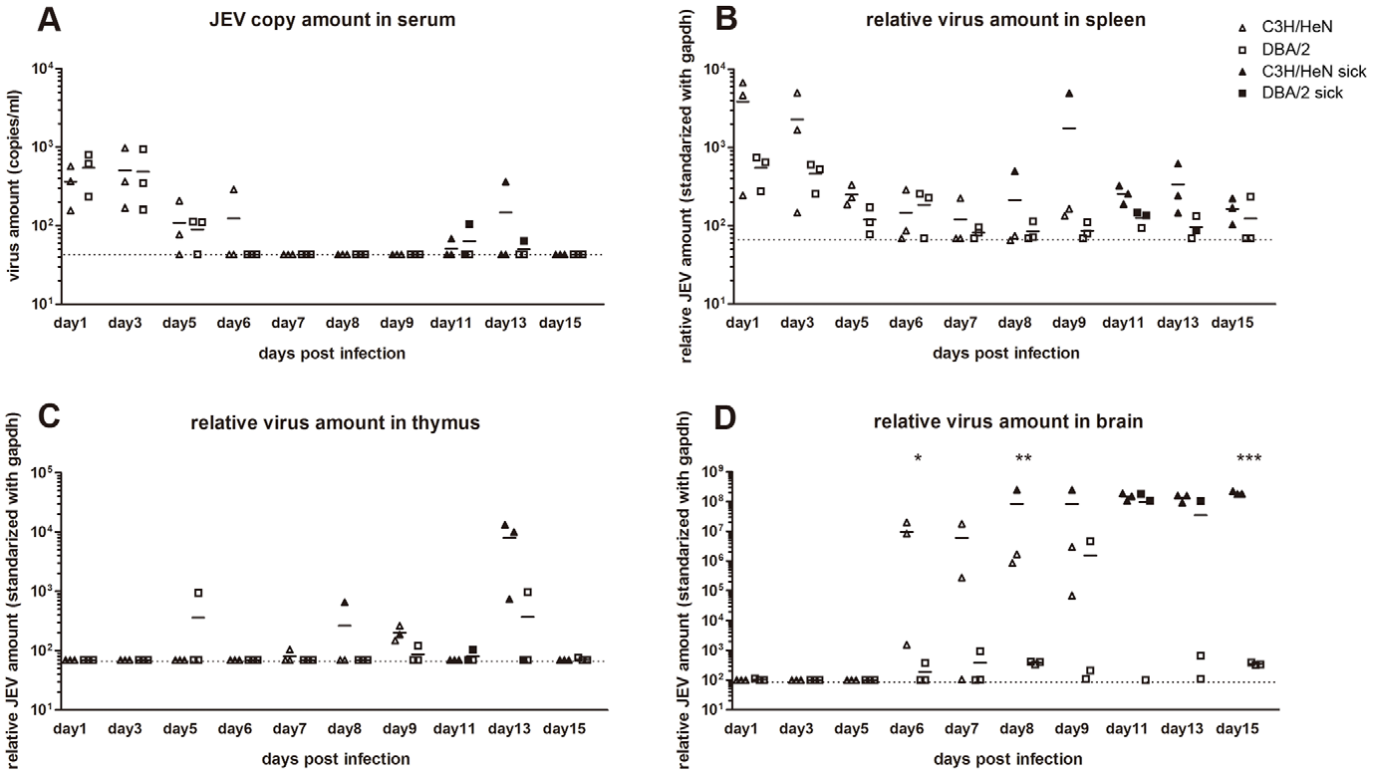
Viral loads in sera and organs of C3H/HeN and DBA/2 mice infected with JEV SA14 virus. Serum (A), spleen (B), thymus (C) and brain tissues (D) were tested for JEV RNA by real-time RT-PCR. The viral RNA loads in the tissues were standardized with GAPDH RNA and those in the sera were calculated as RNA copies per ml. The detection limit of RNA copies/ml is 10^1.6^ for serum and of relative JEV/GAPDH (JEV/GAPDH x10^8^) ratio is 10^1.8^ for the spleen and thymus tissues and 10^2^ for brain tissue. The point values at the dotted line are recorded as undetectable. The white triangles are displayed for C3H/HeN and the white squares for DBA/2 values. Symptomatic mice were recorded as dying mice and presented with black symbols. The lines indicate the average amount of three samples studied the same day. *, p<0.05; **, p<0.01; ***, p<0.001 for comparisons between the two mouse strains.

Mild viremia was detected from days 1 to 3 p.i. with a mean value of approximately 10^3^ RNA copies/ml in all mice tested, which subsequently decreased to 10^2.1^ copies/ml at day 5 p.i. (Fig. 2A). The threshold of RNA detection of the test was 10^1.6^ copies/ml. The viral RNA loads in the sera were higher than the amount of virus injected, which indicated that the viral load detected did not correspond to the inoculum. Low levels of viral RNA were detected on days 11 and 13 p.i., but only in one sick mouse (Fig. 2A).

Spleen, thymus, and brain tissues were sampled from both infected and mock-infected mouse strains on different days p.i. and RNA was extracted. Relative amounts of JEV RNA was compared by real-time RT-PCR and standardized with the RNA of the house keeping gene, GAPDH (Fig. 2B, C). The baseline of the ratio JEV RNA/GAPDH RNA was 10^1.8^ to 10^−8^, which corresponded to the limit of detection of JEV RNA. Low viral amounts were detected at days 1 to 3 p.i. in spleen tissue, which subsequently decreased during the remaining course of the infection (Fig. 2B), whereas JEV was undetectable in thymus tissues from the majority of mice (Fig. 2C).

JEV RNA was detected in brain tissues of C3H/HeN mice starting on day 6 and on day 9 p.i. in the DBA/2 strain (Fig. 2D). Increasing amounts of viral RNA were observed before mice showed symptoms of infection. From day 11 p.i., both mouse strains that showed severe symptoms had similar JEV RNA loads. At 15 days p.i. and after, none of the brains from tested from either strain of mouse without symptoms contained viral RNA.

### Histology and immunohistochemistry in infected-mouse brain

JEV E protein or NS1 protein was detected from 6 days p.i. in neuronal cells of the cortex and thalamus in the brain tissues of C3H/HeN infected mice and in the hippocampus of dying mice (Fig. S1G-I). Progressive accumulation of JEV-positive neurons and perivascular inflammatory responses were observed in both mouse strains. However, a delay of 2 to 3 days occurred in pathological alteration in infected DBA/2 mice compared to infected C3H/HeN mice. Figure S1 (B, C, E, F) shows perivascular and cortical inflammatory infiltration and pathological changes after 9 days p.i. in C3H/HeN mice, whereas milder alterations were observed on the same day in the infected DBA/2 strain. The number of the infected neurons was lower in DBA/2 than in C3H/HeN (Fig. S1E, F). However, the brains of dying mice showed similar histopathological changes and estimated numbers of infected neurons in both mouse strains, which mirrored viral load data.

### Quantification of cell markers and inflammatory molecules in brain tissue of infected mice

**Figure 3:**
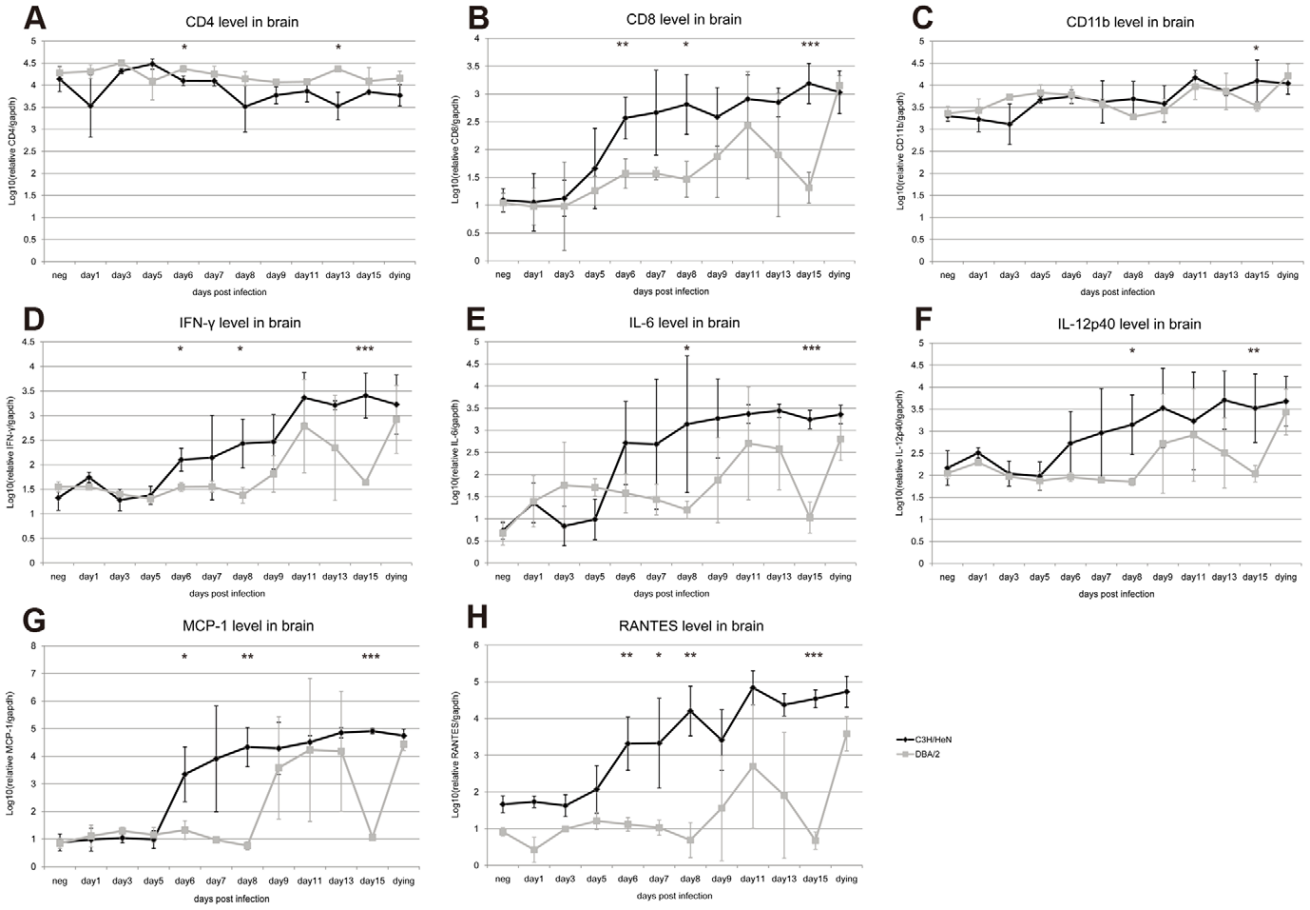
Relative mRNA levels of antigen markers specific for inflammatory responses in the brain of infected mice. Relative levels of mRNA from CD4, CD8 and CD11b cell markers (A, B, C) were calculated. Cytokine mRNA levels were tested for IFN-γ, IL-6, IL-12p40 (D, E, F) and chemokines MCP-1 and RANTES (G, H). All mRNAs were standardized with GAPDH and presented in log10 means ± standard deviation for three mice tested on the same day. mRNA expression in C3H/HeN mouse brain tissue is represented by black spots and in DBA/2 mouse brain by light grey squares. All samples were tested three times. *, p<0.05; **, p<0.01; ***, p<0.001 for comparisons between the two mouse strains.

Histological observations showed a major infiltration of inflammatory cells in the brain tissues of JEV-infected mice (Fig. S1) without the possibility of identifying their phenotypes due to the lack of immuno-reactivity of commercially available antibodies with cellular antigens fixed in PFA-treated specimens. To determine the phenotypes of the cells infiltrating the brain, the mRNA concentrations of CD4, CD8b, and CD11b cells were examined by real-time RT-PCR. The mRNA values were standardized by using the ratio of mRNA markers to GAPDH mRNA. A remarkable increase of CD8 mRNA was observed in JEV-infected mice (Fig. 3B). In C3H/HeN mice, CD8 mRNA increased significantly from day 6 p.i., while in some DBA/2 mice, high values of CD8 mRNA were observed at day 9 p.i. The expression of CD8 mRNA in dying mice increased approximately 100-fold compared to the negative controls of both strains (p<0.0001) (Fig. 3B). On the contrary, slightly lower levels of CD4 mRNA were observed in JEV-infected mice than the mock-infected mice of both strains (p = 0.041) (Fig. 3A). No significant differences were observed in CD11b mRNA levels between the two mouse strains and a 6-fold increase was observed in dying mice compared with mock-infected mice (p<0.0001) (Fig. 3C). A large variation in the values of CD8 and CD11b mRNAs was observed amongst the three infected mice tested each day, which may reflect the differences observed in the appearance of symptoms and outcomes of infected mice.

The cytokine and chemokine mRNAs started to accumulate in brain tissue from day 6 p.i and peaked after day 9 p.i. in the C3H/HeN strain. In DBA/2 mice, the mean cytokine mRNA levels started to increase on day 9 and peaked between days 11 and 13 p.i. (Fig. 3). A large variation in cytokine and chemokine mRNA concentrations among the three mice tested daily was also observed from days 6 to 9 p.i. in the C3H/HeN strain and days 9 to 13 p.i. in DBA/2 mice, which was due to the different infection statuses of the sacrificed mice (Fig. 3D-H). In general, similar levels of increased cytokine mRNAs were observed in dying mice in both strains. IFN-γ and IL-12p40 mRNA levels increased more than 30-fold over the negative controls (p<0.0001) (Fig. 3D, F), whereas the level of IL-6 reached about a 500-fold increase (p<0.0001) (Fig. 3E). The chemokines, including MCP-1 and RANTES, play an active role in recruiting leukocytes to inflammatory sites and both slightly increased at day 6 p.i. in C3H/HeN mice. The increased values of up to 100 times those of the negative control likely followed viral neuroinvasion and reached up to 1000 times the control values in dying mice (p<0.0001) (Fig. 3G, H). MCP-1 and RANTES remained at low levels in asymptomatic DBA/2 mice, whereas dying mice displayed similar high amounts of mRNA among the two strains (Figure 3G).

### Cytokine and chemokine analyses in mouse sera

**Figure 4:**
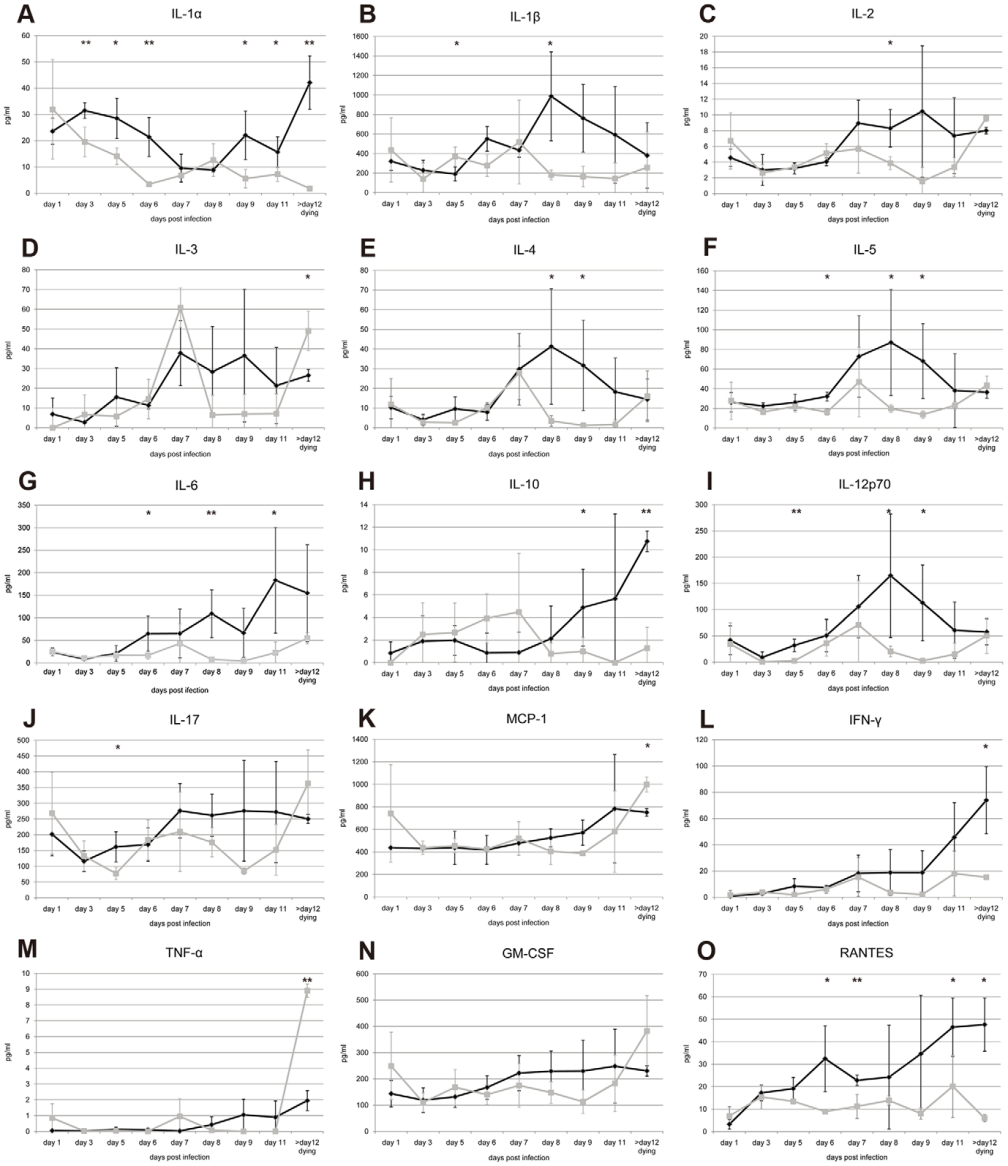
Cytokine and Chemokine levels in serum of JEV-infected mice. Cytokines were measured using the Q-Plex™ Mouse Cytokine array and were compared with the standard samples in the kit. Black spots at indicated times signifies the mean values of three C3H/HeN mice and grey spots indicate those of DBA/2 mice. Standard deviation bars are presented for the three mice tested on the same day. MIP-1α that showed only a negligible amount is not presented. *, p<0.05; **, p<0.01 for comparisons between the two mouse strains.

The levels of multiple cytokines and chemokines (IL-1α, IL-1β, IL-2, IL-3, IL-4, IL-5, IL-6, IL-10, IL-12p70, IL-17, MCP-1, IFN-γ, tumor necrotic factor (TNF)-α, macrophage inflammatory protein (MIP)-1α, granulocyte macrophage colony-stimulating factor (GM-CSF) and RANTES were tested in infected mouse sera from days 1 to 11 p.i. and in three dying mice after 12 days p.i.. The cytokine titers varied considerably between the three individuals tested daily for each mouse strain, which could be associated with a variation within the time frame needed for disease manifestation. Less variation was usually observed in the titers of dying mice compared with those surviving, presumably because mice were at the same stage of infection and disease. IL-1α was the only cytokine that showed a consistent decrease in its mean level from 30 pg/ml to 3 pg/ml at day 6 p.i. in the DBA/2 strain and to 9 pg/ml at day 8 p.i. in C3H/HeN mice (Fig. 4A). However, IL-1α increased to a mean value of 40 pg/ml in very sick C3H/HeN mice, but remained at low levels in dying DBA/2 mice. Increased titers of IL-1β, IL-2, IL-4, IL-5, IL-6, IL-12p70, IL-17, IFN-γ and GM-CSF were observed from day 6 or 7 to day 8 p.i. in C3H/HeN mice, whereas no remarkable increase of cytokine titer appeared in sera of infected DBA/2 mice (Fig. 4). Cytokine IL-3 showed a sharp increase at day 7 p.i. in the DBA/2 mice, whereas only a slight increase was observed in C3H/HeN mice, but with no statistical significance (Fig. 4D). IL-10 showed an early modest increase in DBA/2 mice from day 3 to day 7, which then dropped to normal concentrations at day 8 before any symptoms of disease occurred, whereas its titer started to increase only at day 8 in sick C3H/HeN mice (Fig. 4H). TNF-α was barely detectable in either mouse strain, but showed a sharp increase in dying DBA/2 mice (Fig. 4M).

### JEV-specific antibodies elicited in infected mice

**Figure 5:**
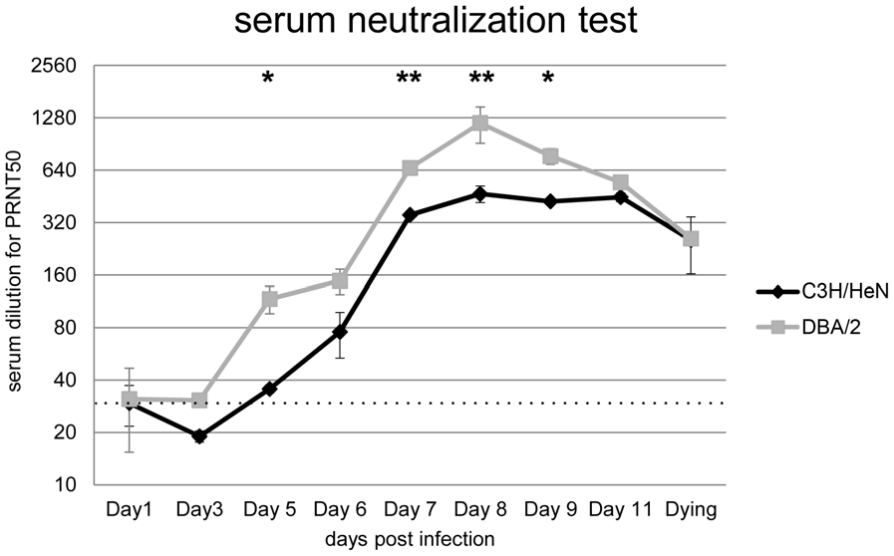
JEV specific antibody responses in mouse sera during the time course of infection. The plaque reduction neutralization test (PRNT) in BHK cells was used to quantitate anti-JEV neutralizing antibodies. *, p<0.05; **, p<0.01 for comparisons between the two mouse strains. The dotted line indicates the background from negative mouse sera.

In order to compare the host's humoral immune responses in the two mouse strains at early stages of infection, JEV-specific antibodies were tested from days 1 to 11 p.i. Although sera were heated before testing to inactivate any virus and complement factors, the first dilution (1∶20) of mock-infected mouse sera and of sera from infected mice collected at days 1 and 3 p.i. showed non-specific 50% decrease of the virus plaques. Sera from infected DBA/2 mice exhibited neutralizing titers of 1∶80 as early as day 5 p.i. that later reached 1∶1,280 at day 8 p.i., whereas neutralizing antibody concentrations of infected C3H/HeN mice were significantly less at day 5 p.i. and peaked at a lower titer (1∶480) (p = 0.0451) (Fig. 5).

### JEV replication in primary myeloid cells and fibroblast cultures from different strains of mice

**Figure 6:**
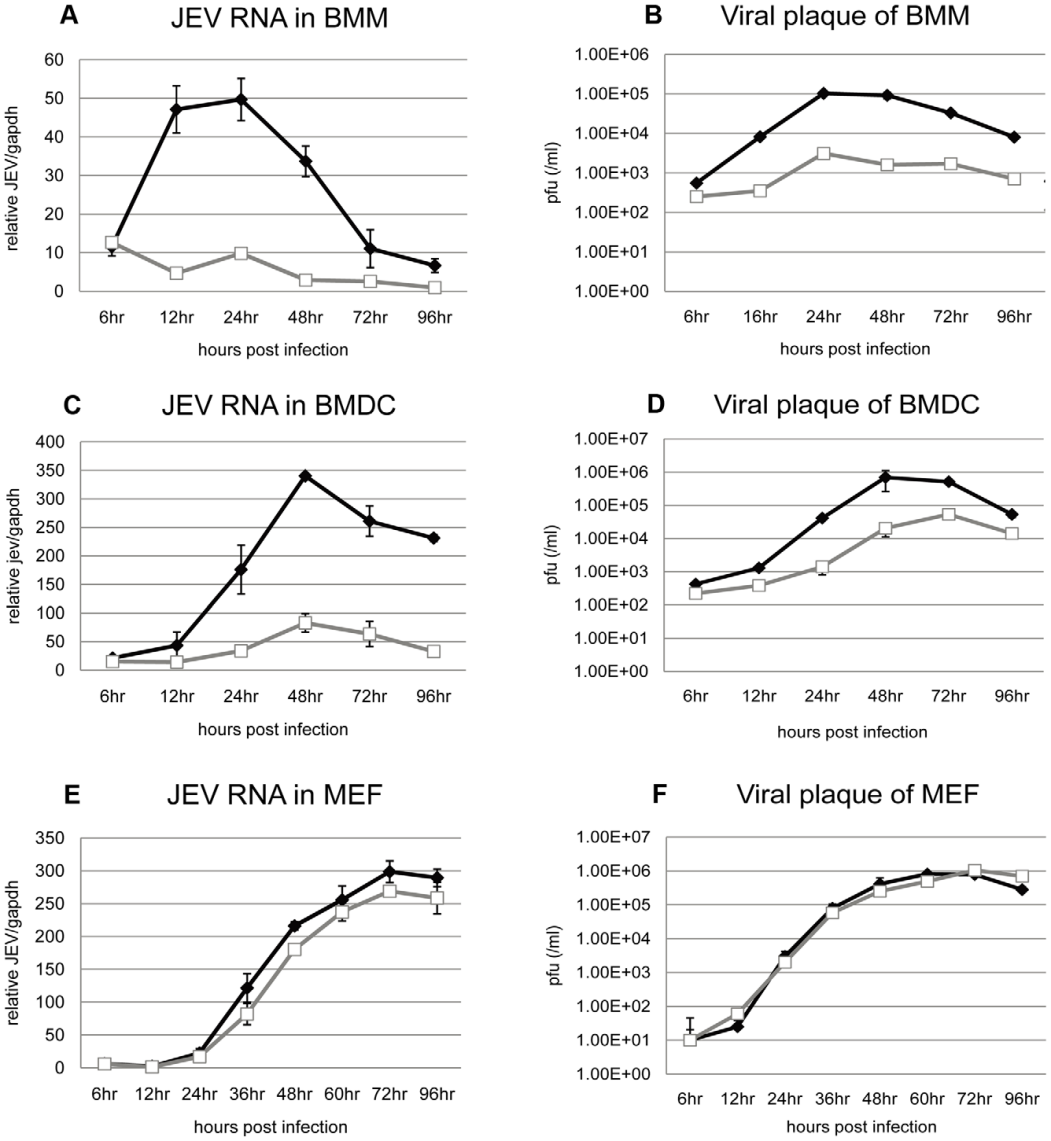
JEV replication in primary cell cultures. BMM (A, B), BMDC (C, D) and MEF (E, F) were isolated from C3H/HeN and DBA/2 mouse strains and infected with a moi of 1 pfu/cell for BMM and BMDC, or a mo of 0.1 pfu/cell for MEF. Total RNA was extracted at indicated times after infection. Intracellular JEV RNA was calculated by Q-RT-PCR (A, C, E). Samples were normalized by GAPDH. Infectious virus titers in cell supernatants were evaluated by viral plaque assay (B, D, F). Dark spots represent samples from C3H/HeN mice and white squares represent samples from DBA/2 mice. Experiments were performed in quadruplicate for at least three independent experiments.

Since macrophages and dendritic cells are potential early target cells for flaviviral infection [12], [20], [33] and are antigen presenting cells (APC) that trigger host innate and adaptive immune responses, we compared JEV infections of BMM and BMDC isolated from each mouse strain. The cells were infected at a moi of 1 pfu/cell and tested for RNA viral replication (Fig. 6A, C) and titration in cellular supernatant (Fig. 6B, D). No morphological changes or apoptotic events occurred in infected cells up to 4 days p.i. (data not shown). Both BMM and BMDC were permissive to JEV replication. In BMM, JEV started to replicate as early as 12 hr p.i. and reached a peak of viral production at 24 hr p.i. In BMDC, viral replication started at 24 hr p.i. and the cells released ten times more viruses in the cell supernatants at 48 hr p.i. compared to BMM. When susceptibilities to JEV infection was compared in myeloid cells from both mouse strains, the cells from C3H/HeN mice sustained higher viral replication and produced higher titers than those from DBA/2 mice (p<0.0001). Remarkably, the viruses remained at very low replication levels in DBA/2-derived BMM, with a virus titer almost two log scales lower than what was observed in C3H/HeN-derived cells (Fig. 6B).

In order to verify whether differences observed in APC susceptibility to JEV infection between the two mouse strains was reproduced in other cells, MEF were infected with JEV at a moi of 0.1 pfu/cell. MEF were highly permissive to the virus and similar concentrations of JEV RNA copies tested by real-time RT-PCR were detected in infected MEF of both mouse strains (Fig. 6E). Infectious viruses in the cell culture supernatant were also titrated by viral plaque assay. JEV release started before 24 hr p.i. and reached a peak of over 10^6^ pfu/ml at 60 hr p.i. However, no difference was observed between viral titers produced from infected-cells of the two mouse strains (Fig. 6F).

### JEV infectivity rates of myeloid cells derived from C3H/HeN and DBA/2 mice

Differences of JEV replication were observed in myeloid cells but not in MEF derived from the two mouse strains as illustrated in the data mentioned above. In order to evaluate the ratio of infected cells, polyclonal antibodies against the JEV E protein were used to label infected cells and cells were subsequently observed by immunofluorescence at 48 hr p.i. The amount of infected MEF was similar for both mouse strains (Fig. S2). On the contrary, the numbers of BMM- and BMDC-infected cells derived from C3H/HeN mice were higher than those of DBA/2-derived cells (Fig. S2). FACS analysis was carried out to more accurately estimate the percentage of infected BMM by labeling the infected cells with an anti-NS1 polyclonal antibody (Figure S2B, C). Consistent with the data obtained by immunofluorescence, the ratio of BMM-infected cells was low and the peak of infection occurred at 48 hr. The number of JEV-infected cells derived from C3H/HeN was 11.4±2.2%, 24.3±3.6%, and 14.9±2.4% at 1, 2 and 3 days p.i., respectively, whereas it was 4.7±0.9% (p = 0.049), 8.6±1.5% (p = 0.029) and 2.1±0.6% (p = 0.017) for DBA/2-derived cells (Fig. S2). Noticeably, the F4/80 membrane antigen was presented at higher amounts on the BMM surface within the time of infection, which resulted from cellular activation during JEV infection. Repeated low staining of JEV antigens in infected BMDC using different antibodies did not provide interpretable FACS data analysis.

### Cytokine and chemokine release is replication-dependent in JEV-infected primary cells in mice

**Figure 7:**
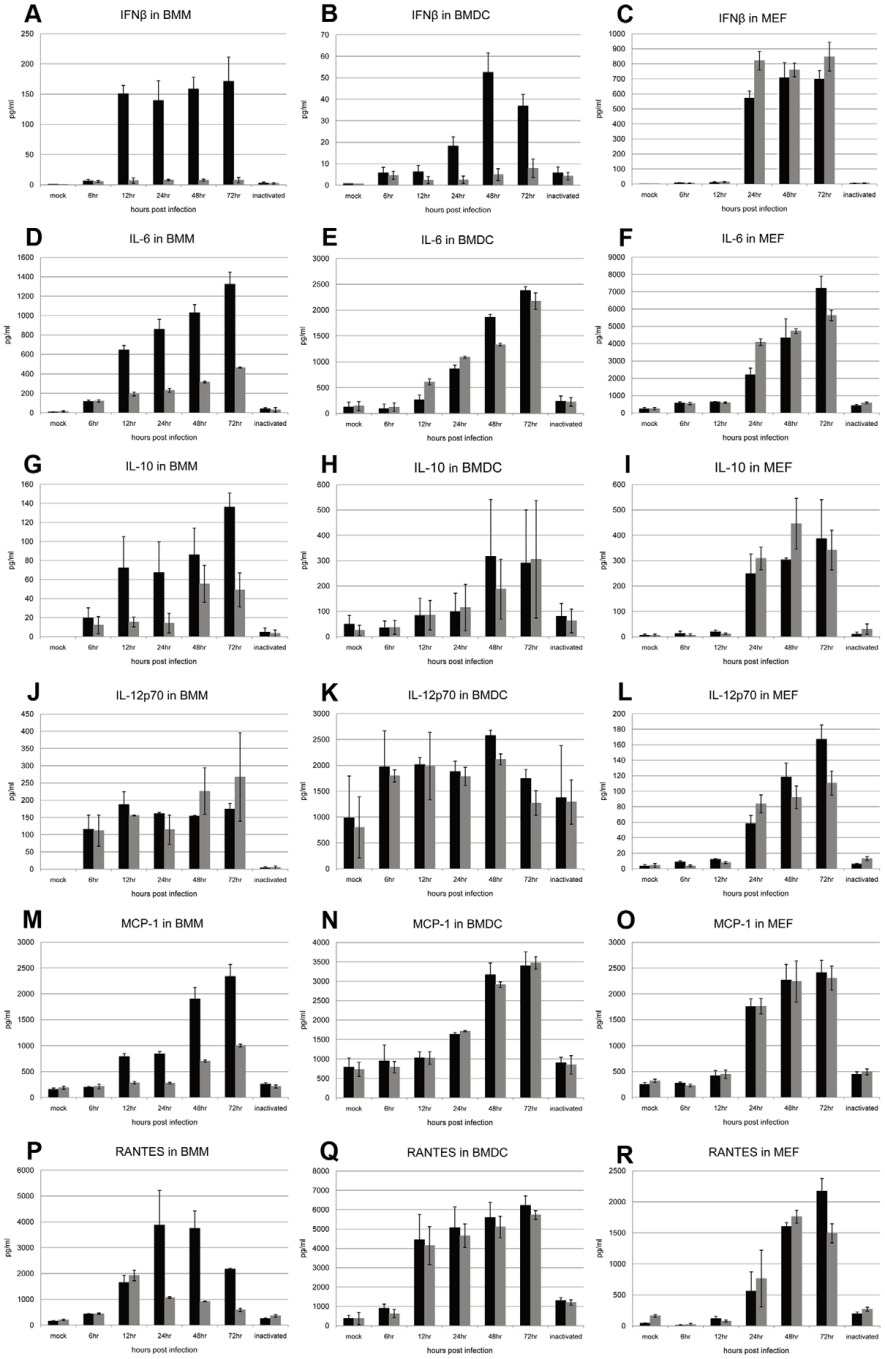
Cytokines produced by BMM and MEF infected with JEV. The profiles of cytokine production including IFN-β, IL-6, IL-10, IL-12p70, MCP-1 and RANTES were quantified at indicated times post-infection in supernatants of JEV-infected BMM (A, D, G, J, M, P), BMDC (B, E, H, K, N) and MEF (C, F, I, L, O, R). The means ± standard deviation was calculated from the values of three mice. For negative controls, cytokine production was tested from cell supernatants 48 hr with no virus or after incubation with UV-inactivated virus (inactivated). Black columns represent values of samples derived from C3H/HeN-derived cells and grey columns represent those from DBA/2-derived cells.

To investigate whether innate immunity contributes to the modulation of viral replication, IFN-β, IL-6, IL-10, IL-12p70, MCP-1 and RANTES were examined in MEF, and in BMDC and BMM myeloid cells (Fig. 7). UV-inactivated JEV was used as a control of viral binding to cells but not for replication within the cells. The inflammatory molecules tested in cells incubated with inactivated viruses showed only slightly higher levels than those observed in uninfected cells. No significant differences in cytokine or chemokine values were observed in infected MEF of either mouse strain, which reflected the similar JEV replication and production rates observed in Fig. 6 (Fig. 7C, F, I, L, O, R). Higher concentrations of cytokines and chemokines were observed in JEV-infected BMM from CH3/HeN mice compared to cells derived from the DBA/2 strain (Fig. 7A, D, G, M, P), which was consistent with the viral replication (Fig. 6). Only IL-12p70 showed similar values (Fig. 7J). Notably, the level of IFN-β remained very low in DBA/2 infected BMMs although it was highly activated in C3H/HeN-derived cells (Fig. 7A). In contrast, although IFN-β production was much higher in BMDC from C3H/HeN mice than from DBA/2, the levels of the other cytokines and chemokines were similar. In addition, the time of appearance and the level of cytokine and chemokine production showed large variations among the two APC types. Soluble markers appeared earlier but at lower titers in BMM than in BMDC (Fig. 7), in accordance with the JEV replication rate (Fig. 6).

## Discussion

Mice show similar features of acute encephalitis compared to humans after JEV infection and have been used as a model of flaviviral pathogenesis [17]. However, biological and virological markers indicating the severity of Japanese encephalitis in mice and humans remain unclear. Previous studies did not show significant differences in JEV production in peripheral organs or in serum among strains of mice [25], [26]. Comparisons of tissue tropism, viral replication and kinetics in C3H and B6 mice, which differ in the number of acute WNV disease and death rates, indicate that tissue tropism is similar for the two strains of mice [28]. Differences in mouse susceptibilities to the flavivirus Banzi was also observed among C3H/He and C3H/RV strains, which was not dependent on resistance of tissues to viral replication [34]. Taking advantage of different susceptibilities among mouse strains to JEV, the objectives of this study were to identify biological markers that could be related to differences in survival rates of highly susceptible (C3H/HeN) and less susceptible (DBA/2) mice infected by peripheral routes with JEV.

Replication in spleen tissue and viremia were reported in mice infected with JEV [9], [10], [35]. In this study, JEV RNA was found in low amounts in serum and spleen tissues before day 5 p.i. and no replication was observed in thymus tissue. However, no significant difference was observed in viral RNA in serum or spleen tissue of either mouse strain. Not all mice had viral neuroinvasion with lower susceptibility in DBA/2 than in C3H/HeN suggesting differences in activated host response in the periphery, and only mice with brain infection showed symptoms and death within the following two days. Interestingly a difference was observed between JEV and WNV infections and mouse recovery after brain infection. Mice with different susceptibilities to WNV and infected by subcutaneous route all showed brain infection but morbidity and mortality differed between the mouse strains, suggesting different capacities of virus clearance [33]. Pro-inflammatory cytokines, including IFN-γ, IL-6 and IL-12p40, were produced in the brain when the JEV was replicating in neurons of both C3H/HeN and DBA/2 mouse strains. This finding was consistent with previous observations in mice infected with flaviviruses [18], [21], [36], [37]. MCP-1 and RANTES, which were reported to play a role in recruiting immune cells to infected tissue, increased up to 1000 fold in the brains of dying mice of both strains. It is possible that these cytokines were produced in the brain by residing in activated cells or were recruited by the viral replication that further regulated the inflammatory response [19], [21]. Accumulation of cytokines and chemokines in the CNS may accentuate progression of encephalitis instead of restricting viral replication [21], [38]. Other studies have suggested a protective role of proinflammatory cytokines and chemokines against flaviviral encephalitis [14], [33]. A study carried out in humans had reported a significant increase in IL-6, RANTES and IL-8 in the CSF of patients infected with JEV compared to controls [39]. Whether the profile of cytokine production in human encephalitis will predict the disease outcome requires further study. In all, the viral replication and host responses in the CNS did not differ between the resistant and susceptible mouse strains once the virus had entered into the brain. This is consistent with similar mortality rates observed in C3H/HeN and DBA/2 mice infected by intracerebral routes, which suggests that the clinical differences observed are not correlated with a distinct neurovirulence or inflammatory response in the brain, but rather with virus-host interactions prior to viral neuroinvasion.

Whether early events occurring during the incubation phase could trigger innate or acquired immune responses, which subsequently could contribute to CNS invasion of or protection against a neurotropic flavivirus, remains poorly understood. It was proposed that early TNF-α production during WNV mouse infection could be a potential factor responsible for an increase in the permeability of the BBB [40], [41]. Dying DBA/2 mice had higher levels of TNF-α, IL-3 and MCP-1 than C3H/HeN mice although they had lower levels at the onset of the disease, which may reflect a late activation of those cytokine pathways (Fig. 4). However, in this study, the TNF-α level in blood remained almost undetectable in both JEV-infected mouse strains at early stage, suggesting that this cytokine did not contribute to the viral entry into the brain. Moreover, none of the cytokines tested were significantly increased in sera before the virus invaded the CNS, suggesting that subtle changes or other factors may have a role in vascular permeability and viral entry into the brain [42]. A higher mortality in WNV-infected IFN-γ (-/-) and TCR-Δ (-/-) mice suggested that IFN-γ plays a protective role in the early stages of infection before viral dissemination to the CNS [43], [44]. However, an increased level of IFN-γ was observed in JEV-infected C3H/HeN mouse sera, which increased slightly at day 7 p.i. and reached maximum values when mice were symptomatic, indicating a minor role in the modulation of JEV entry into the CNS. The amount of cytokines including IL-6, IL-4, IL-12p70 and RANTES, started to increase in C3H/HeN mouse sera only after day 6 p.i., when the virus was already detected in brain tissue. It was observed that in C3H/HeN mice, the level of proinflammatory cytokines IL-6 and RANTES correlated with viral replication levels and the severity of disease and was detected in the CSF and sera of flaviviral-infected patients [14], [39], [45]. No significant difference was found in the amount of cytokine transcriptome levels in spleen or thymus tissues among mock-infected, moderately sick or severely ill mice at any time (data not shown), indicating very low to no viral detection in these organs. However, a recent study has found an mRNA up-regulation of proinflammatory cytokines and chemokines, including IL-1, IL-6, IFN-γ, MCP-1, and IFN-inducible transcription factors, in both spleen and brain tissues from JEV-infected BALB/c mice [46]. Mice used in that study were only one week old, and their immunological immaturity to contain virus replication may explain differences observed in response of lymphoid tissues to infection with JEV.

Correlation between the presence of neutralizing antibodies and protection against viral diseases is generally well accepted in flavivirology. Passive protection of mice infected with JEV [47] or WNV [33], [48], [49] clearly indicates a crucial role in humoral immunity to viral diffusion and fatal outcomes. In C3H/HeN, JEV was detected in the brain tissue of infected mice at day 6 p.i., which appeared at the same time as neutralizing antibodies. Conversely, no significant viral load was detected in DBA/2 infected brain tissues before day 9 p.i. where neutralizing antibodies were detected at day 5 p.i. and at higher levels than in the C3H/HeN strain. These differences may have contributed to the delay and partial prevention of JEV neuroinvasion in DBA/2 mice.

MEF have been extensively used to study the host immune response towards virus infection [44] and the genetic susceptibility (Flv*^S^*) to flaviviral infection referred to as the IFN type I pathway knockdown mutation, which leads to higher replication levels in MEF [23], [24], [43]. Therefore, MEF derived from both mouse strains were infected with JEV to compare viral replication and innate immune responses. JEV replicated at the same rate in both mouse-derived MEF, which was accompanied with similar cytokine and chemokine production [43], [50]. This suggested that there was no apparent modification in MEF tropism and host-cell innate immune responses to JEV infection between strains.

Macrophages and dendritic cells are permissive to JEV infection [10], [20], [51], [52], [53]. In addition, dendritic cells are potential early target cells of flaviviral infection after the bite of an infected mosquito [54], [55], [56]. Macrophagic cells were reported to have a protective role in flaviviral infection, including direct clearance through viral uptake from circulation without viral progeny production or apoptosis of infected cells [8], [57], [58]. However, some studies have reported that macrophages act as reservoirs that carry viruses from the periphery to the CNS [11], [53]. In this study, quantitation of intracellular viral RNA and of viral particles released from infected cells showed that C3H/HeN-derived myeloid cells were significantly more susceptible to JEV-infection than those derived from the DBA/2 strain. The viruses replicated efficiently in myeloid cells of C3H/HeN strains, while almost no or much lower replication was detected in BMM and BMDC of DBA/2 mice. The immunofluorescence and FACS analyses showed differences in the number of infected CH3H/HeN-derived myeloid cells compared to DBA/2-derived cells. Differences in cell infectivity by flaviviruses may be due to (co)receptor polymorphisms. Mannose receptors and DC-SIGN or DC-SIGNR receptors on macrophages are utilized by DENV and WNV, respectively [59], [60]. However, macrophage cell receptors for JEV remain unknown, and FACS analysis of DC-SIGN, DC-SIGNR or mannose receptor of BMDC and BMM of the two mouse strains did not show significant difference in the level of those receptors (data not shown). After 1 h of incubation with JEV, quantitation of viral RNA binding or internalization in cells indicated similar viral uptakes by BMM from both mouse strains (data not shown). It is possible that macrophagic cells from DBA/2 mice may modulate cell infection through partial viral degradation in the phagosomic pathway, an anti-viral process that has been described in DENV infection [8], [58].

Surprisingly, almost no IFN-β was induced in myeloid cells from DBA/2 mice, although it was produced at high levels in MEF. Whether this cytokine knockdown in myeloid cells plays a role in the reduced amount of virus and DBA/2 mouse susceptibility to JEV compared to the C3H/HeN strain requires further study. The IL-6, IL-10, MCP-1 and RANTES production paralleled viral replication in BMM from both mouse strains. Although viral replication was lower in BMDC from DBA/2 compared with C3H/HeN mice, the levels of these cytokines increased to similar levels, which suggested that differences observed in the viral replication during JEV infection may be correlated more to susceptibility of APCs from C3H/HeN mice to JEV infection than to activation of these cytokines. Whether higher APC susceptibility was directly or indirectly responsible for the more efficient neuroinvasion in C3H/HeN mice is difficult to address at this time without a clear understanding of the pathway used by JEV to cross the BBB.

In conclusion, the comparative *in vivo* mouse infection did not show clear-cut differences in viral or soluble markers of innate immunity among highly susceptible and less susceptible mouse strains. However, earlier and greater amounts of neutralizing antibodies could have limited the invasion of JEV in the brain in less susceptible mice. Moreover, a lower JEV infectivity capacity was observed in bone marrow-derived primary macrophages and dendritic cells of the less susceptible mouse strain. These differences represent biological factors which may contribute to partial protection against neuroinvasion in DBA/2.

## Supporting Information

Figure S1 **Histopathological observation of brains from JEV- infected mice.** Photomicrographs of staining was taken in mock mice (A, D), sick mice of the C3H/HeN strain (B, E) and one of the DBA/2 strain at day 9 post-infection (C, F). Compared with the negative control, the brain tissues of both sick mice displayed inflammatory cell infiltration, but the infiltration of DBA/2 was milder. Hematoxylin and eosin staining was preformed for brain tissue (A, B, C, X100). Rabbit anti-JEV NS1 polyclonal antibodies were used for detection of the virus in the brain. The positive staining of neurons in C3H/HeN mice was more severe than in the DBA/2 strain (D, E, F, x200). Representative photos were shown in different brain regions including the cerebral cortex (G), thalamus (H) and hippocampus (I) for one sample of C3H/HeN mice at day 15 post-infection, x200 magnification.
(TIF)

Figure S2 **Infection ratio of MEF, BMM and BMDC.** C3H/HeN- or DBA/2-derived MEF and myeloid cells (BMM and BMDC) were infected with JEV at a moi of 0.1 pfu/cell and 1 pfu/cell, respectively, and fixed 48 hr p.i. The number of cells expressing JEV E-protein was checked by immunofluorescence using anti-E polyclonal antibodies (A). Photomicrographs (magnification x40) are representative for three wells per group and repeated for four independent experiments. FACS analysis was performed on JEV-infected BMM at 0, 24, 48 and 72 hr p.i. using JEV anti-NS1 polyclonal antibody (B). The mean F4/80+NS1+ percentage is presented graphically (C). *, p<0.05 for comparisons between the two mouse strain.
(TIF)
