# Supplementary figures and images for: Mice with Different Susceptibility to Japanese Encephalitis Virus Infection Show Selective Neutralizing Antibody Response and Myeloid Cell Infectivity
Source: PLoS One. 2011 Sep 16;6(9):e24744. doi: 10.1371/journal.pone.0024744 (PMC3174963; doi:10.1371/journal.pone.0024744)

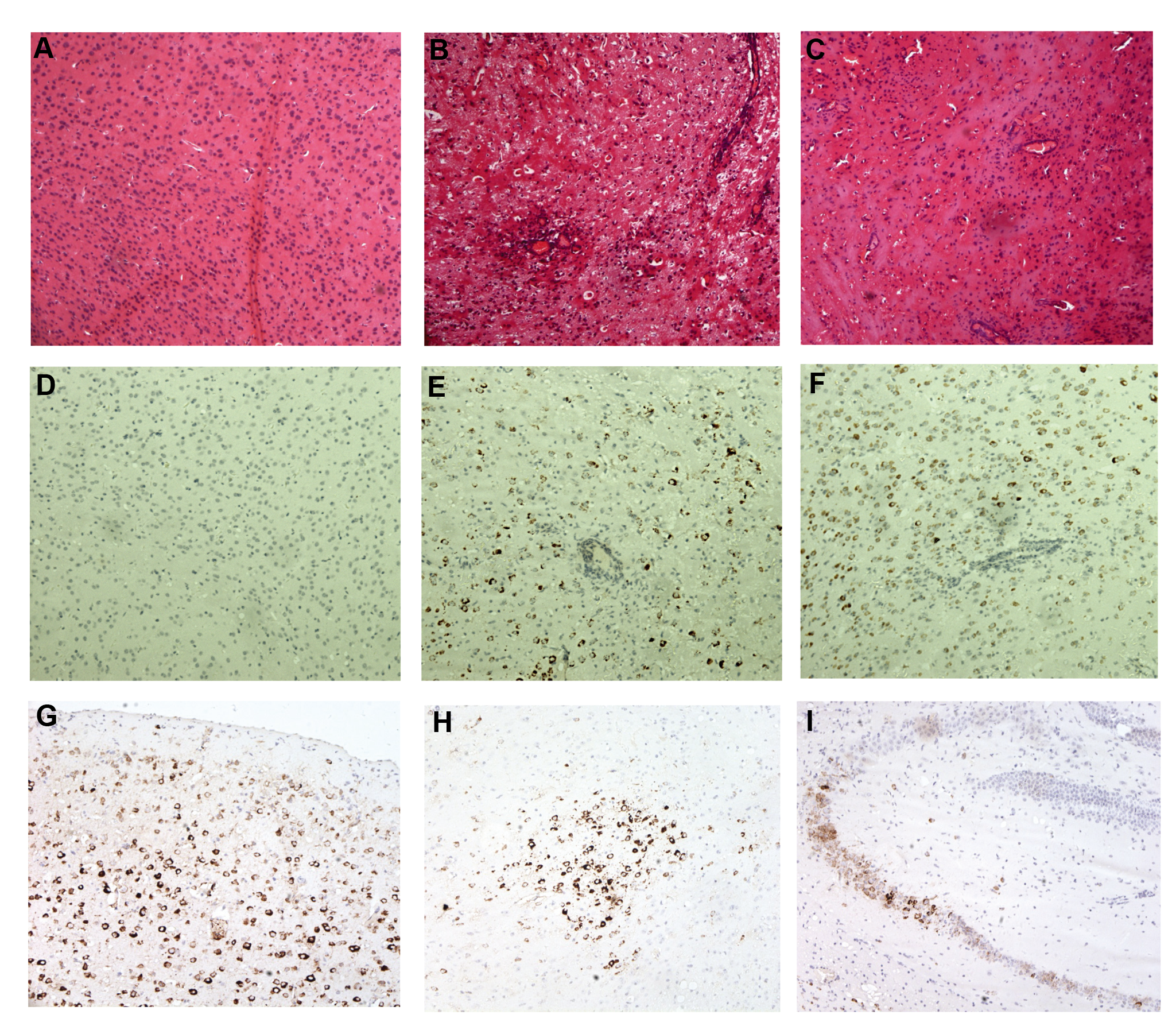

Supplement: Figure S1 — Histopathological observation of brains from JEV- infected mice. Photomicrographs of staining was taken in mock mice (A, D), sick mice of the C3H/HeN strain (B, E) and one of the DBA/2 strain at day 9 post-infection (C, F). Compared with the negative control, the brain tissues of both sick mice displayed inflammatory cell infiltration, but the infiltration of DBA/2 was milder. Hematoxylin and eosin staining was preformed for brain tissue (A, B, C, X100). Rabbit anti-JEV NS1 polyclonal antibodies were used for detection of the virus in the brain. The positive staining of neurons in C3H/HeN mice was more severe than in the DBA/2 strain (D, E, F, x200). Representative photos were shown in different brain regions including the cerebral cortex (G), thalamus (H) and hippocampus (I) for one sample of C3H/HeN mice at day 15 post-infection, x200 magnification. (TIF) [file pone.0024744.s001.tif]

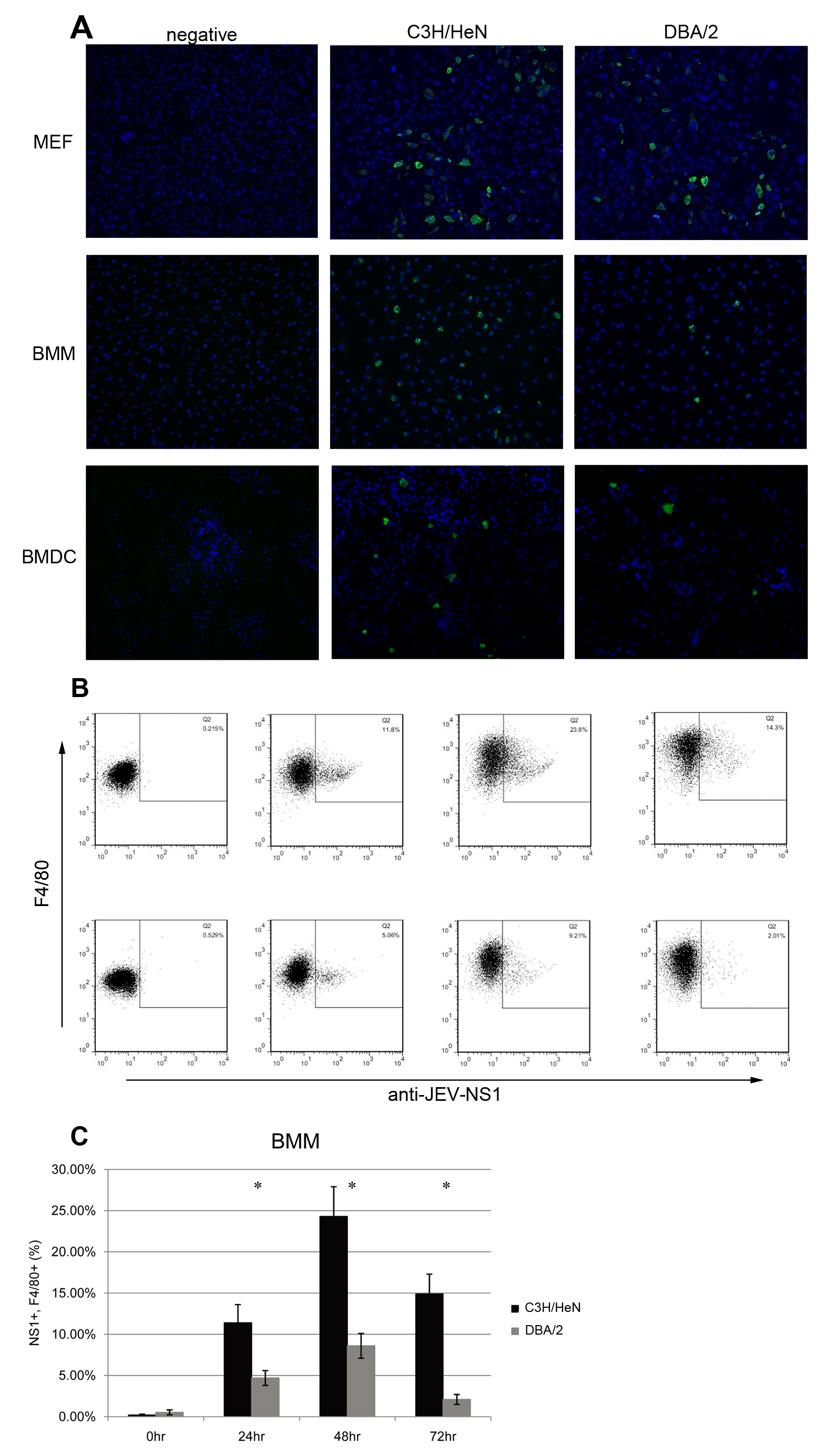

Supplement: Figure S2 — Infection ratio of MEF, BMM and BMDC. C3H/HeN- or DBA/2-derived MEF and myeloid cells (BMM and BMDC) were infected with JEV at a moi of 0.1 pfu/cell and 1 pfu/cell, respectively, and fixed 48 hr p.i. The number of cells expressing JEV E-protein was checked by immunofluorescence using anti-E polyclonal antibodies (A). Photomicrographs (magnification x40) are representative for three wells per group and repeated for four independent experiments. FACS analysis was performed on JEV-infected BMM at 0, 24, 48 and 72 hr p.i. using JEV anti-NS1 polyclonal antibody (B). The mean F4/80+NS1+ percentage is presented graphically (C). *, p<0.05 for comparisons between the two mouse strain. (TIF) [file pone.0024744.s002.tif]
